# Nursing management of pulmonary mucormycosis with skin damage secondary to amphotericin B colloidal dispersion: a case report

**DOI:** 10.3389/fmed.2025.1723602

**Published:** 2026-02-10

**Authors:** Yu Liu, Hao Wang, Shiyao Wang

**Affiliations:** Department of Respiratory and Critical Care Medicine, Shengjing Hospital of China Medical University, Shenyang, China

**Keywords:** adverse effects, amphotericin B, extravasation, immunocompromised, mucormycosis, skin damage

## Abstract

**Introduction:**

Mucormycosis is a catastrophic fungal infection in immunocompromised patients and has a high mortality rate. Amphotericin B colloidal dispersions have demonstrated clinical efficacy in the treatment of mucormycosis; however, there is limited literature on the management of severe skin damage secondary to the extravasation of amphotericin B colloidal dispersions.

**Case presentation:**

A 36-year-old diabetic woman with severe pulmonary mucormycosis developed skin damage on her left foot after 34 days of amphotericin B colloidal dispersion treatment via a peripheral vein. The medication was switched to a central vein, and nursing interventions, including silver alginate ion dressing and saline wet compress, successfully managed the injury. By day 155, the skin had completely healed with minimal residual pigmentation.

**Conclusion:**

This case report describes the management of a severe skin injury caused by amphotericin B colloidal dispersion extravasation in an immunocompromised patient, carried out by bedside nurses.

**Relevance for clinical practice:**

This study presents an effective management strategy for skin damage caused by amphotericin B colloidal dispersions extravasation, emphasizing timely intervention and optimizing care for patients receiving prolonged or high-dose therapy.

## Highlights

- Mucormycosis is a severe fungal infection in immunocompromised patients, treated with amphotericin B colloidal dispersion.
- A diabetic patient developed skin damage from amphotericin B colloidal dispersions extravasation, which was managed with nursing interventions.
- The skin injury healed completely by hospital day 155 with minimal pigmentation.

## Introduction

The number of immunocompromised individuals has increased markedly in recent years (1). In these patients, pulmonary infections are a key factor influencing prognosis (2). Pulmonary mucormycosis poses a significant threat, particularly in those with poorly controlled hyperglycemia and diabetic ketoacidosis. Mortality rates for pulmonary mucormycosis are extremely high, ranging from 48 to 87% (3). Timely administration of amphotericin B colloidal dispersions, the preferred medication, is crucial for improving patient survival (3).

Although amphotericin B colloidal dispersions have significant antifungal activity, they are associated with notable adverse reactions. Clinically observed adverse reactions include nephrotoxicity, electrolyte imbalance, infusion reactions, and phlebitis (4). However, there is a lack of studies regarding the substantial skin damage caused by the extravasation of amphotericin B colloidal dispersions during intravenous infusion. Such adverse reactions pose significant challenges for both the treatment of mucormycosis and the provision of adequate nursing care. In this study, we report the nursing management of a patient who developed severe skin damage secondary to the extravasation of amphotericin B colloidal dispersions.

## Case presentation

The case protocol was approved by the ethics committee of the authors’ hospital, and the patient provided written informed consent for publication of the case details and associated images.

A 36-year-old woman was admitted to our department with cough, expectoration, and dyspnea. One month prior to admission, she presented with a cough with white sputum of unknown cause. She was treated with several oral antibiotics; however, her symptoms did not improve significantly. Two weeks later, the patient exhibited worsening dyspnea. The patient, presenting without fever, dizziness, headache, nausea, vomiting, diarrhea, rash, photosensitivity, recurrent oral ulcers, or alopecia, reported poor appetite and sleep. The 24-h urine output was approximately 300 mL, with no significant weight loss noted, suggesting possible acute kidney injury or reduced effective circulating volume. Past medical history was negative for coronary heart disease, hypertension, diabetes mellitus, hepatitis, tuberculosis, prior surgery, trauma, or blood transfusion. The patient reported no known drug or food allergies. Social history revealed a 9-year smoking history of approximately 10 cigarettes per day and no alcohol use. There was no contributory family history of hereditary diseases. There was no evidence of malignancy or an immunocompromised state at the time of admission. Due to severe respiratory failure, the patient promptly underwent life-saving endotracheal intubation and mechanical ventilation in the emergency department. Contrast-enhanced chest computed tomography revealed thickening and narrowing of the bronchial walls in the upper and middle segments of the right lung, along with patchy shadows in the right upper lobe and an irregular high-density area near the right lung hilum. Bronchoscopy revealed swelling of the mucosa in both the left and right main bronchi with yellow, purulent secretions attached. Moreover, the bronchi of the right upper and middle lobes were narrowed, and a fistula was visible on the inner side of the right main bronchus. Histopathological examination of the bronchial mucosa revealed inflammatory necrotic tissue, accompanied by granuloma formation. Culture results of sputum and alveolar lavage fluid revealed the growth of *Rhizopus arrhizus*. Thus, the patient was diagnosed with pulmonary mucormycosis and transferred to the medical intensive care unit (MICU) for further treatment. The patient denied having a history of DM but presented with hyperglycemia upon admission, with venous blood glucose levels as high as 27.8 mmol/L. Her glycated hemoglobin A1c (HbA1c) level was 13.5%.

**Figure 1:**
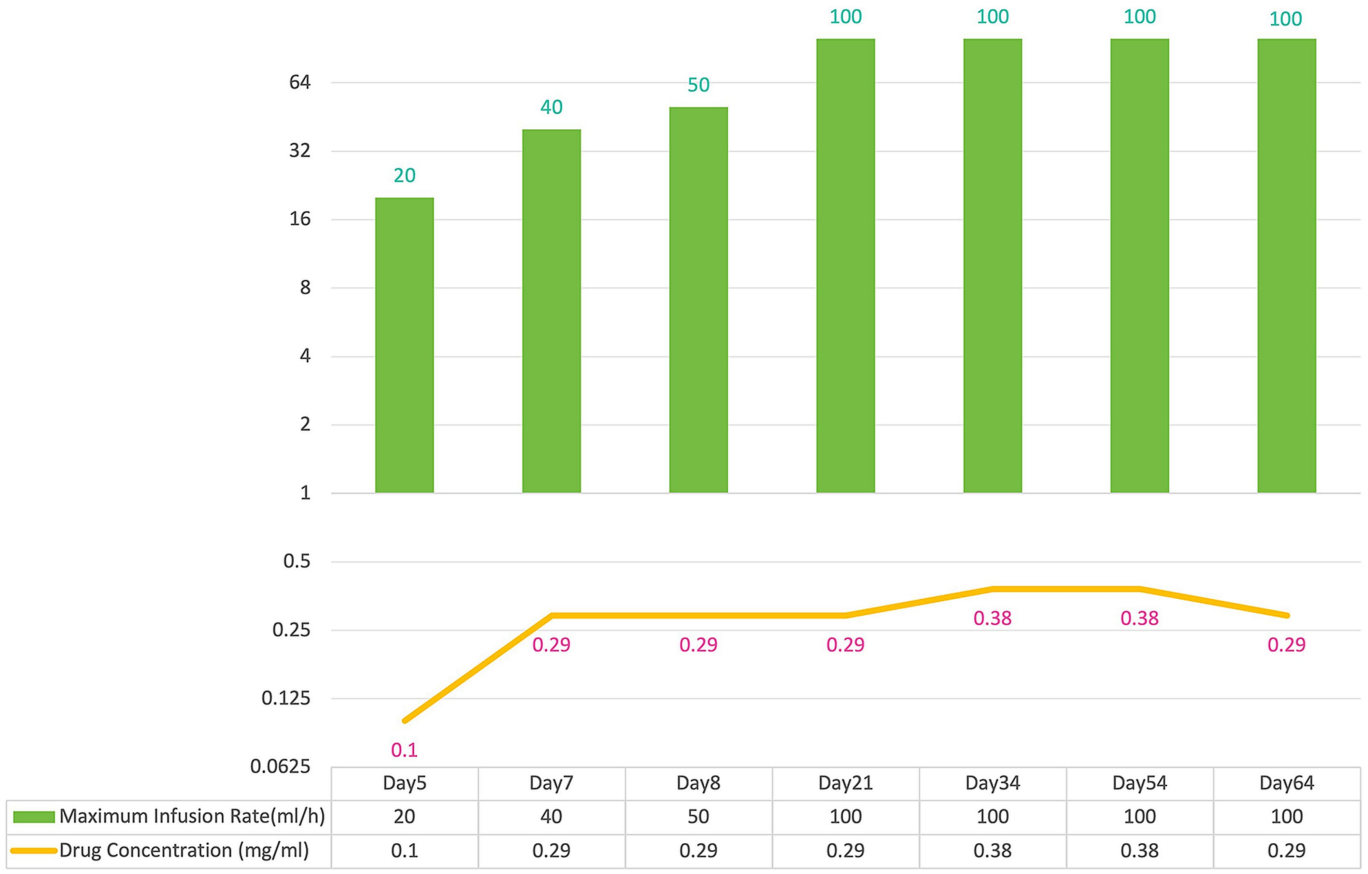
Infusion rates and drug concentrations of amphotericin B colloidal dispersion during the treatment process.

**Figure 2:**
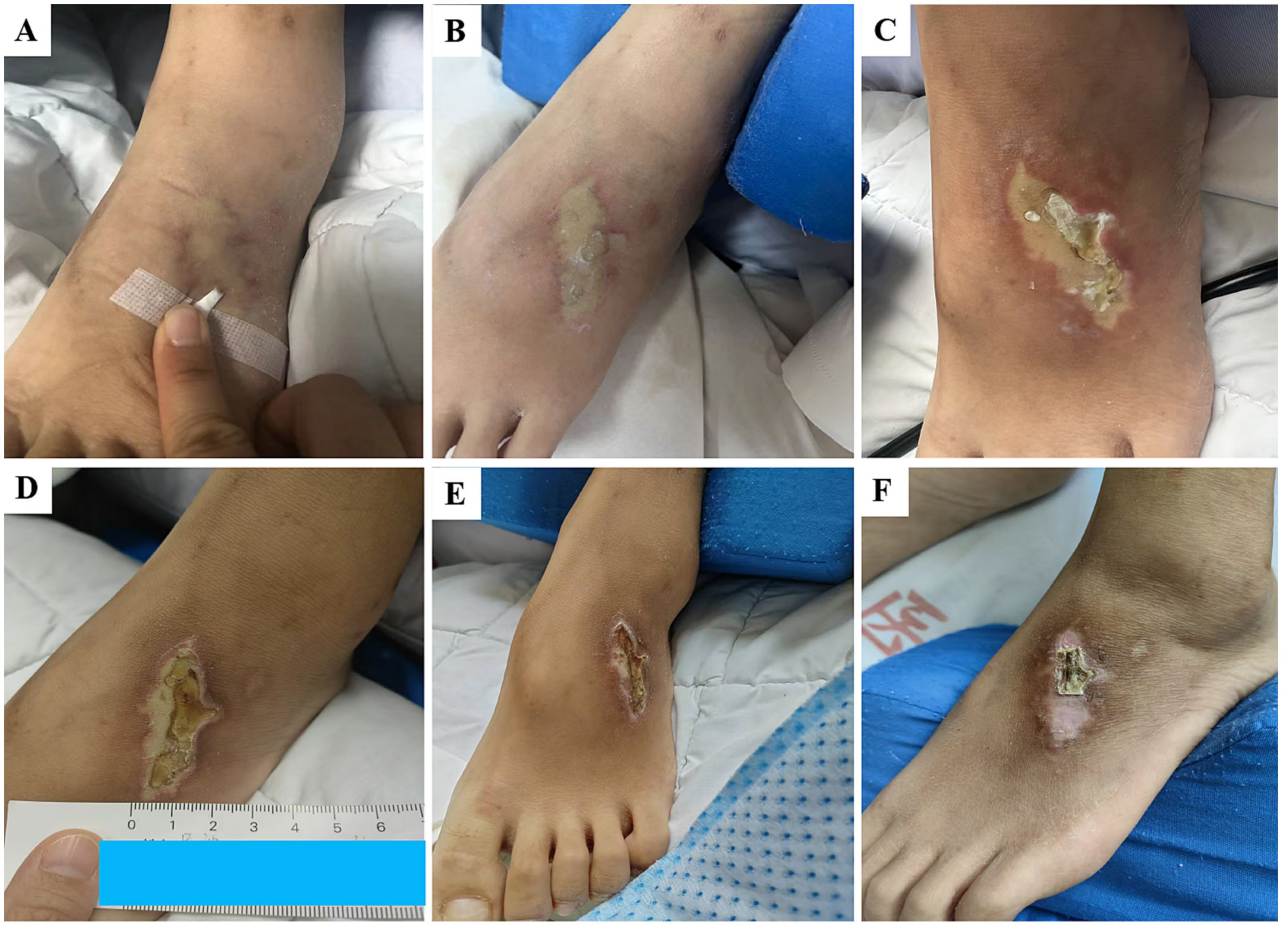
Cutaneous changes in the patient’s foot during the administration of amphotericin B colloidal dispersion. **(A)** Pallidity of the skin within a 2.5–5 cm radius around the injection site on the left dorsal foot during amphotericin B colloidal dispersion infusion (hospital day 53). **(B)** Gradual deepening of the color of the damaged skin to yellow with the presence of scattered serous blisters (hospital day 55). **(C)** Persistent yellow coloration of the damaged skin; the blisters have dried and fallen off, leaving an area of ulceration (hospital day 66). **(D)** Lightened color of the affected skin became lighter in color during treatment with a dressing incorporating silver alginate ions and saline compression; the base surface and rupture area of the skin are dry and crusted (hospital day 79). **(E)** Lightened color of the damaged skin; the base surface and rupture area continue to be dry and crusted (hospital day 92). **(F)** Further increase in the pale area of skin; the base surface of the skin and the ruptured scabs are dried, with some of the blisters falling off (hospital day 105).

The patient was treated with an amphotericin B colloidal dispersion via intravenous injection and amphotericin B colloidal dispersion inhalation. Other treatments included mechanical ventilation, meropenem (500 mg, administered every 8 h via intravenous drip), and tigecycline (50 mg, administered every 12 h via intravenous drip), antibiotics for secondary *Acinetobacter baumannii* infections, bronchoscopy, insulin for DM, and enteral nutrition. The patient developed severe shock secondary to severe infection. To maintain stable blood pressure, noradrenaline and dopamine were infused through the patient’s central venous line. Given the complete utilization of the patient’s central venous access, amphotericin B colloidal dispersions were administered via a peripheral venous route, with the dose starting at 25 mg/day and then gradually increasing. The nursing team strictly controlled the infusion speed, which was 20 mL/h during the first 2 h, 30 mL/h during the third hour, and finally 40 mL/h thereafter. On hospital day 5, the dose of amphotericin B colloidal dispersion was increased to 150 mg (3 mg/kg) daily (Figure 1). After treatment with this dose for 27 days, the patient showed no significant remission, presenting with hyperthermia and requiring high-concentration oxygen via an invasive ventilator to maintain oxygenation. Furthermore, bronchoscopy showed diffuse stenosis of both bronchi with mucosal swelling and purulent secretions. Therefore, on hospital day 34, the dose of amphotericin B colloidal dispersion increased to 200 mg daily. During the amphotericin B colloidal dispersion infusion, the nurse noticed pallidity in a 2.5-cm to 5-cm area of skin around the injection site on the left dorsal foot on hospital day 53 (Figure 2A). At this stage, with the pulmonary mucormycosis infection still active, the multidisciplinary team (MDT) decided to establish a dedicated central venous access (a femoral venous catheter was inserted). This decision was driven by the need to continue amphotericin B colloidal dispersions as first-line therapy while mitigating the significant risk of extravasation, thereby ensuring safe and reliable intravenous delivery. The patient was hemodynamically unstable, which contraindicated any surgical intervention. Following a multidisciplinary surgical consultation, the consensus was to prioritize medical support and stabilization, deferring any surgical plans. Given the lesion’s location in a sensitive area with minimal subcutaneous tissue and its close proximity to neurovascular structures, a conservative approach without debridement was adopted. The team applied 2.5 g of magnesium sulfate 10 mL compression once daily and topical mucopolysaccharide polysulfate (drug specification:100 g cream contains 300 mg mucopolysaccharide polysulfate) twice daily, along with multisulfated polysaccharides to alleviate the subcutaneous tissue edema. The color of the damaged skin gradually deepened to yellow, and scattered serous blisters were observed (Figure 2B). Three days later, the team used a sterile syringe to aspirate the aggravated serous blisters. However, the damaged skin remained yellow, and the surface of the skin where the dried blisters had fallen off showed ulceration (Figure 2C). On hospital day 66, the nursing team decided to treat the damaged skin using a combination of a silver alginate ion dressing and saline compression. Following this treatment, the patient’s skin color gradually lightened, and the ulcerated areas dried and formed scabs (Figures 2D–F).

**Figure 3:**
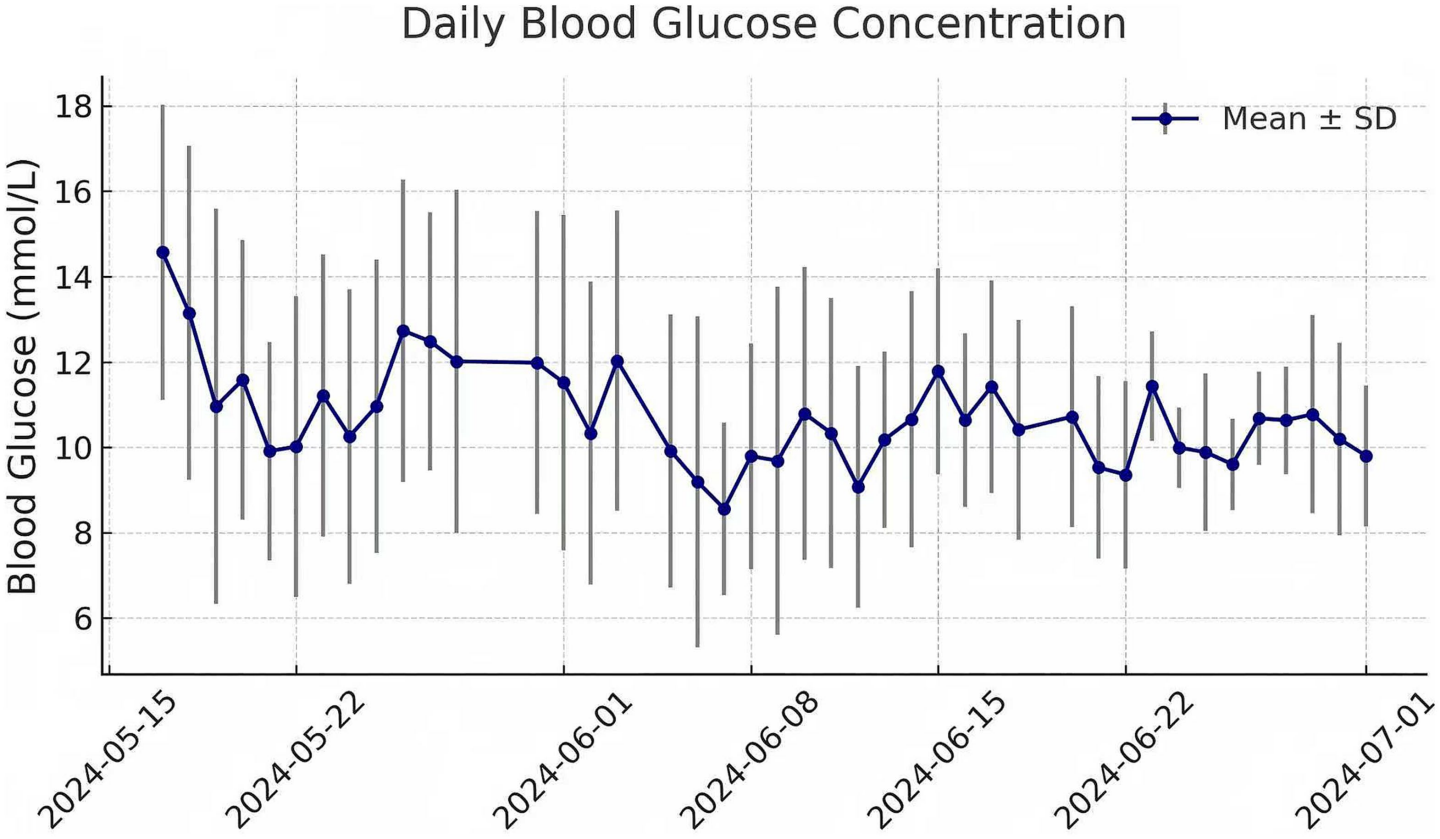
Analysis of the daily mean and standard deviation of blood glucose concentrations. The figure illustrates daily variations in blood glucose concentrations over a specific time period. Each data point represents the mean blood glucose level for a given day, with error bars indicating the standard deviation (SD) to reflect daily fluctuations. The curve shows an overall trend in blood glucose control, highlighting periods of elevated or more stable glucose levels. Data were collected at multiple time points throughout each day and then averaged to compute the daily mean.

Concurrently, the nursing team also closely monitored and controlled the patient’s blood sugar levels to promote healing of the damaged skin (Figure 3). Additionally, the patient experienced recurrent vomiting due to gastroparesis; enteral nutrition can be administered through a retained jejunal feeding tube to increase the patient’s energy supply.

After the above procedures were administered by the nursing team, the patient’s clinical condition gradually stabilized. Invasive mechanical ventilation was discontinued on day 109. On hospital day 146, a repeat CT and bronchoscopy showed reduced sizes of the occupying lesions and remission of airway stenosis. The skin on the left foot was fully healed, exhibiting only minimal residual pigmentation. The patient was successfully discharged from the hospital.

## Discussion

### Clinical application and adverse effects of amphotericin B colloidal dispersions and their lipid formulations

Although the incidence of adverse effects has decreased since the introduction of amphotericin B colloidal dispersions, such events remain difficult to avoid in clinical practice, particularly among critically ill patients who require amphotericin B colloidal dispersions due to the failure of other antifungal therapies (5). In these situations, vigilant monitoring for adverse effects during the administration of amphotericin B colloidal dispersions and the implementation of appropriate interventions are essential for improving treatment outcomes in these patients.

**Figure 4:**
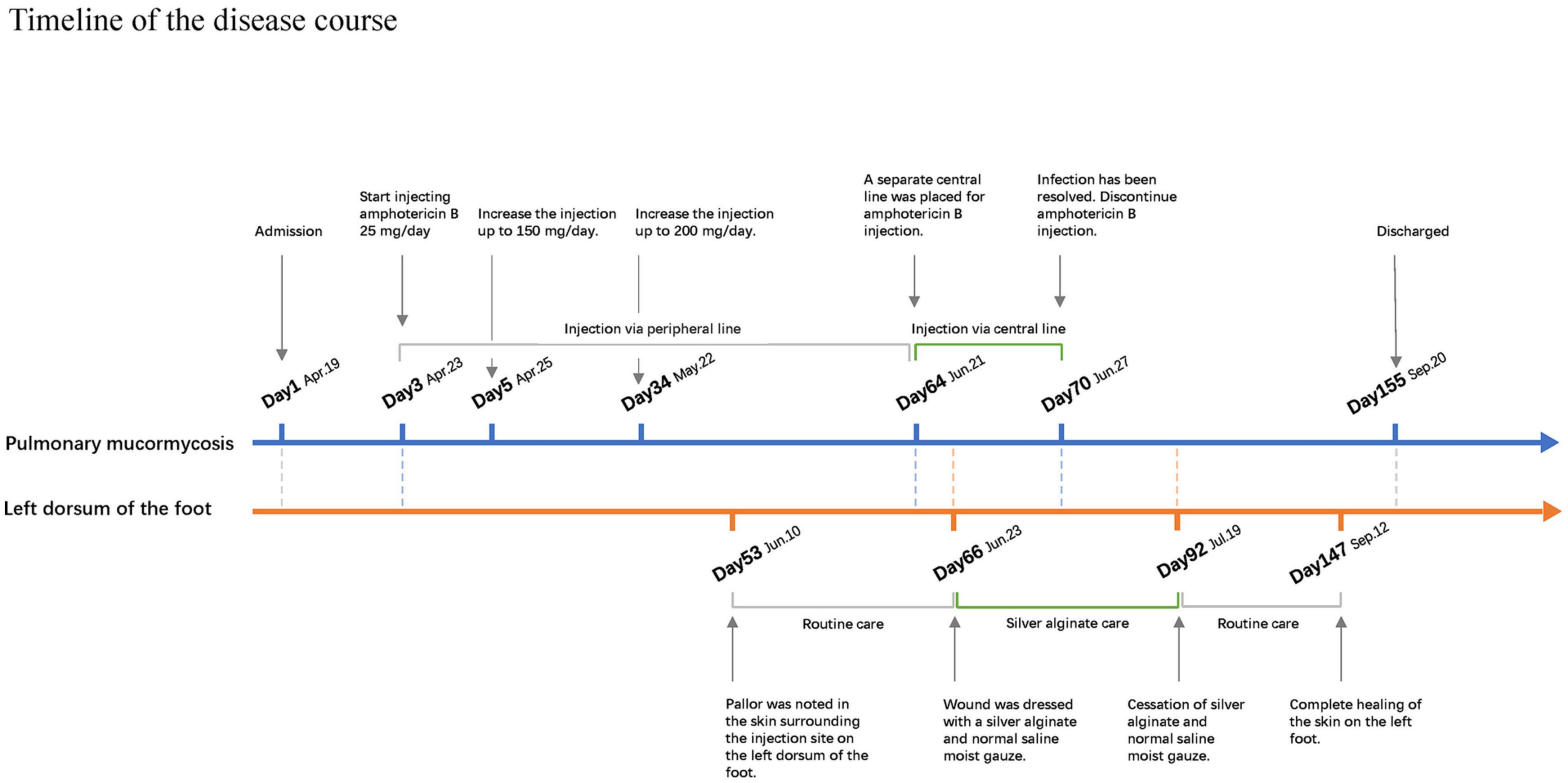
Timeline of the disease course and the treatment regimen for pulmonary mucormycosis are independent of the treatment regimen for the left dorsum of the foot.

Figure 4 shows a timeline showing the treatment regimen for pulmonary mucormycosis and the treatment regimen for the left dorsum of the foot injury.

### Potential mechanisms of skin injury caused by the extravasation of amphotericin B colloidal dispersions

In clinical nursing practice, the use of amphotericin B colloidal dispersions is common, and its adverse effects are generally well recognized. However, the extravasation of amphotericin B colloidal dispersions differs from typical phlebitis, making the resulting skin injury less readily detectable. In this case, the skin at the intravenous puncture site initially exhibited subtle pallor without obvious redness, swelling, or induration. Subsequently, the patient developed a necrotic ulcer, expanding outward from the center with surrounding erythema. These findings suggest that amphotericin B colloidal dispersion can cause not only damage to peripheral blood vessels but also necrosis of the overlying skin and subcutaneous tissues.

In this case, the primary cause of amphotericin B colloidal dispersion extravasation leading to skin injury was the highly osmotic nature and direct venous irritant effect of amphotericin B colloidal dispersion-cholesteryl sulfate complex. Therefore, it is recommended that this medication be administered via a central venous line or a peripherally inserted central catheter (PICC).

During the early stage of the patient’s critical illness, multiple vasoactive drugs were administered concurrently to maintain vital signs, as they were essential for circulatory support. These infusions occupied the patient’s only available central venous access—the internal jugular vein. Consequently, with central venous access occupied, the amphotericin B colloidal dispersion-cholesteryl sulfate complex was administered via a peripheral intravenous route. Due to the patient’s poor peripheral vascular condition, venipuncture was challenging, and ultimately, a vein on the dorsum of the left foot was selected for access.

A contributing factor was the patient’s previously undiagnosed and unmanaged hyperglycemia, which had persisted prior to admission. On admission, the patient’s HbA1c level was 13.5%. Chronic hyperglycemia can damage vascular endothelial cells (6), and imbalances in glucose metabolism can activate the polyol pathway, leading to the accumulation of metabolic byproducts that further compromise vascular integrity (6). As a result, the vasopressors used to treat the patient’s septic shock likely induced peripheral vasoconstriction, thereby increasing the risk of skin breakdown (7).

### Nursing interventions and outcome analysis in this case

The patient in this case developed severe skin injury during treatment with amphotericin B colloidal dispersion, presenting a significant challenge for nursing care. Multiple interventions were implemented to improve the patient’s condition. First, the infusion process was closely monitored, and the infusion rate was strictly controlled. Prior to administration, nurses carefully reviewed the drug’s prescribing information to understand its pharmacologic effects and potential adverse reactions. During the infusion, the intravenous puncture site was meticulously inspected every hour to promptly detect any abnormalities.

The infusion rate was maintained in strict accordance with the drug guidelines, which was initially set at 10 to 30 drops per minute and then gradually increased after 2 h, with the total infusion time controlled between 4 and 6 h. In this case, despite strict rate control, extravasation occurred when the drug concentration and fluid volume peaked, suggesting that greater vigilance is required during the dose escalation phases and that early consideration should be given to central venous infusion to minimize the risk of extravasation.

After the skin injury was identified, the nursing team initially applied magnesium sulfate wet compresses to alleviate symptoms; however, the therapeutic effect was limited. Subsequently, treatment was switched to silver alginate dressings combined with normal saline wet compresses. The silver ion dressings effectively managed the wounds with heavy exudate, maintained a moist wound environment, reduced bacterial load, and promoted autolytic debridement of necrotic tissue, which significantly enhanced wound healing (8–11). In this case, this approach demonstrated favorable outcomes in the management of necrotic ulcers caused by extravasation.

Strict glycemic control is essential for mitigating severe infections and promoting the rapid healing of damaged skin (12, 13). The nursing team closely monitored and managed the patient’s blood glucose levels, maintaining them within the target range of 6.1–11.1 mmol/L. Intravenous infusion of low-dose insulin was administered, with the infusion rate adjusted based on monitoring results to achieve effective glycemic control and provide a favorable internal environment for wound healing. In addition, to improve nutritional status and manage complications such as gastroparesis and gastric retention, the nursing team successfully placed a jejunal feeding tube, ensuring adequate enteral nutrient absorption to support recovery from critical illness and enhance wound repair.

### Differential diagnosis: extravasation-induced injury versus diabetic foot ulcer

The injury was strictly confined to the periphery of the intravenous puncture site and exhibited a close temporal association with the infusion of high-concentration medication. This pattern contrasts with diabetic foot ulcers, which typically originate at pressure points or sites of friction.

The documented sequence—rapid evolution from localized pallor to yellowish discoloration, blister formation, and subsequent necrotic ulceration—is more consistent with the trajectory of a chemical insult. This pattern differs markedly from the typically chronic and indolent progression of classic diabetic foot ulcers.

The wound demonstrated progressive healing following a targeted nursing protocol using silver-ion dressings, which are aimed at controlling infection and managing chemically damaged tissue. This favorable response pattern further supports the etiology of extravasation injury rather than a diabetic-related ulcer.

### Comparison of nursing interventions in this case with international standards

According to international nursing guidelines and the Infusion Nurses Society (INS) standards, patients experiencing drug extravasation accompanied by skin necrosis should undergo local debridement to remove necrotic tissue and reduce infection risk (14). However, in this case, the nursing team adopted a conservative approach using silver alginate dressings combined with moist compresses, without performing debridement. This approach ultimately yielded favorable therapeutic outcomes, highlighting the benefits of individualized nursing strategies.

The nursing interventions implemented in this case were largely consistent with international standards (Table 1), particularly with respect to infusion rate control, extravasation monitoring, glycemic management, and nutritional support. However, differences were observed in the choice of administration route and the management of severe extravasation. International guidelines recommend administering highly irritating drugs via central venous access and treating extravasation with immediate cold compresses, limb elevation, drug neutralization, or debridement. In this case, due to limited vascular access, a small peripheral vein was used for infusion, and a conservative treatment approach was taken after extravasation occurred. This finding underscores the need for flexibility in clinical practice, allowing interventions to be tailored to the patient’s specific conditions. The nursing team’s performance in blood glucose control and nutritional support was exemplary, contributing significantly to the patient’s overall recovery and prognosis.

**Table 1:** Comparison of international nursing standards and case practice.

Nursing item	International standard requirement	Case practice
Choice of infusion route	Recommend central venous catheterization to minimize extravasation risk	Peripheral venous infusion through left dorsum pedis vein due to poor vascular access
Infusion rate management	Start at recommended low rate, gradually increase, strictly control maximum rate	Followed recommended rate; extravasation occurred at maximum dose and speed
Extravasation monitoring frequency	High-risk drugs: inspect site every 15–30 min	Hourly inspection of the puncture site
Initial management after extravasation	Immediately stop infusion, elevate limb, apply local cold compress	Applied magnesium sulfate wet compress
Management of severe tissue injury	Surgical debridement + moist dressing + surgical intervention if necrosis present	Treated with silver alginate dressing + saline wet compress; no debridement performed
Blood glucose control target	Maintain between 6.1–11.1 mmol/L in critically ill patients	Small-dose intravenous insulin infusion; postprandial glucose <11 mmol/L, fasting <7 mmol/L
Nutritional support strategy	Suspend enteral feeding if gastric retention occurs; consider jejunal tube placement if necessary	Jejunal feeding tube placement after assessing gastric retention; successful enteral nutrition

## Conclusion

This case highlights that, for critically ill patients requiring prolonged or high-dose infusion of highly irritating drugs such as amphotericin B colloidal dispersion—particularly those with comorbidities such as diabetes and poor peripheral vascular status—a proactive vascular access management strategy should be implemented. This includes early establishment and reservation of dedicated central venous access and strict avoidance of infusion through small veins in areas with minimal subcutaneous tissue, such as the dorsum of the hand or foot. Proactive planning is crucial for preventing such severe skin injuries.

This patient had diabetes with poorly controlled blood glucose upon admission, which had already caused vascular endothelial damage. Furthermore, the use of vasoactive drugs in a state of shock exacerbated peripheral vasoconstriction, making the patient’s vascular condition extremely fragile. Under these circumstances, even with standard nursing procedures, infusion of high-concentration, highly irritant amphotericin B colloidal dispersion through small veins on the dorsum of the foot was highly prone to cause severe drug extravasation, leading to skin and subcutaneous tissue necrosis.

Therefore, the key recommendation derived from this case is that the preferred route of administration for amphotericin B colloidal dispersion should be via a central venous line. This approach significantly reduces the direct irritation of the drug on the vascular endothelium and the risk of extravasation, serving as a crucial measure to prevent such severe skin injuries.

## Data Availability

The original contributions presented in the study are included in the article/Supplementary material, further inquiries can be directed to the corresponding author.

## References

[1] Darmon M Ranzani OT Azoulay E. Focus on immunocompromised patients. Intensive Care Med. (2017) 43:1415–7. doi: 10.1007/s00134-017-4857-2, 28597035

[2] Ramirez JA Musher DM Evans SE dela Cruz C Crothers KA Hage CA . Treatment of community-acquired pneumonia in immunocompromised adults: a consensus statement regarding initial strategies. Chest. (2020) 158:1896–911. doi: 10.1016/j.chest.2020.05.598, 32561442 PMC7297164

[3] Steinbrink JM Miceli MH. Mucormycosis. Infect Dis Clin N Am. (2021) 35:435–52. doi: 10.1016/j.idc.2021.03.009, 34016285 PMC10110349

[4] Miceli MH Chandrasekar P. Safety and efficacy of liposomal amphotericin B for the empirical therapy of invasive fungal infections in immunocompromised patients. Infect Drug Resist. (2012) 5:9–16. doi: 10.2147/IDR.S22587, 22294858 PMC3269132

[5] Moen MD Lyseng-Williamson KA Scott LJ. Liposomal amphotericin B: a review of its use as empirical therapy in febrile neutropenia and in the treatment of invasive fungal infections. Drugs. (2009) 69:361–92. doi: 10.2165/00003495-200969030-00010, 19275278

[6] Clyne AM. Endothelial response to glucose: dysfunction, metabolism, and transport. Biochem Soc Trans. (2021) 49:313–25. doi: 10.1042/BST20200611, 33522573 PMC7920920

[7] Dunser MW Mayr AJ Tür A Pajk W Barbara F Knotzer H . Ischemic skin lesions as a complication of continuous vasopressin infusion in catecholamine-resistant vasodilatory shock: incidence and risk factors. Crit Care Med. (2003) 31:1394–8. doi: 10.1097/01.CCM.0000059722.94182.79, 12771608

[8] Wong SL Demers M Martinod K Gallant M Wang Y Goldfine AB . Diabetes primes neutrophils to undergo NETosis, which impairs wound healing. Nat Med. (2015) 21:815–9. doi: 10.1038/nm.3887, 26076037 PMC4631120

[9] Nickel B Gorski L Kleidon T Kyes A DeVries M Keogh S . Infusion therapy standards of practice. J Infus Nurs. (2024) 47:S1–S285. doi: 10.1097/NAN.0000000000000532 38211609

[10] Atiyeh BS Costagliola M Hayek SN Dibo SA. Effect of silver on burn wound infection control and healing: review of the literature. Burns. (2007) 33:139–48. doi: 10.1016/j.burns.2006.06.010, 17137719

[11] Geng J Cai Y Lu H Zhang R Tian J Zhang J. Moist dressings in the treatment of pressure injuries: a network meta-analysis. J Tissue Viability. (2023) 32:213–27. doi: 10.1016/j.jtv.2023.03.003, 37012120

[12] Zhang C Zhang S Wu B Zou K Chen H. Efficacy of different types of dressings on pressure injuries: systematic review and network meta-analysis. Nurs Open. (2023) 10:5857–67. doi: 10.1002/nop2.1867, 37386783 PMC10416006

[13] Zhang M Zhao X. Alginate hydrogel dressings for advanced wound management. Int J Biol Macromol. (2020) 162:1414–28. doi: 10.1016/j.ijbiomac.2020.07.311, 32777428

[14] Lee YS Kang SU Lee MH Kim HJ Han CH Won HR . GnRH impairs diabetic wound healing through enhanced NETosis. Cell Mol Immunol. (2020) 17:856–64. doi: 10.1038/s41423-019-0252-y, 31217526 PMC7395134

