# Supplementary material for: Nursing management of pulmonary mucormycosis with skin damage secondary to amphotericin B colloidal dispersion: a case report
Source: Front Med (Lausanne). 2026 Feb 10;12:1723602. doi: 10.3389/fmed.2025.1723602 (PMC12929125; doi:10.3389/fmed.2025.1723602)

Admission

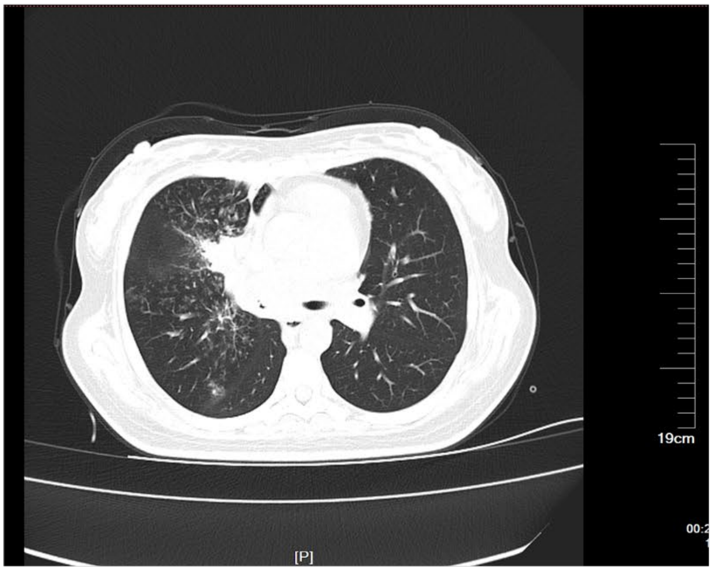

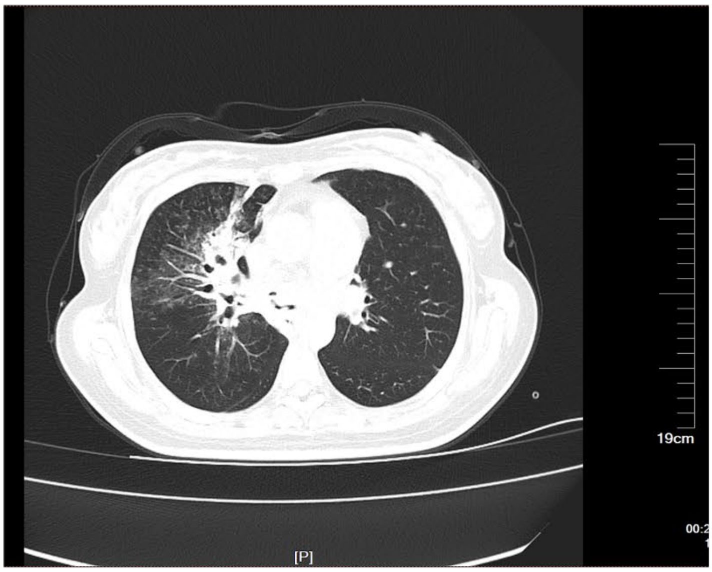

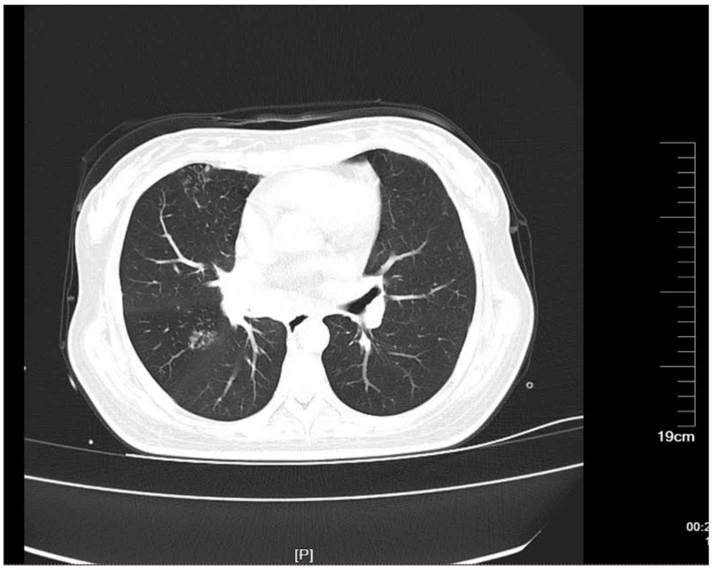

Hospital Day11

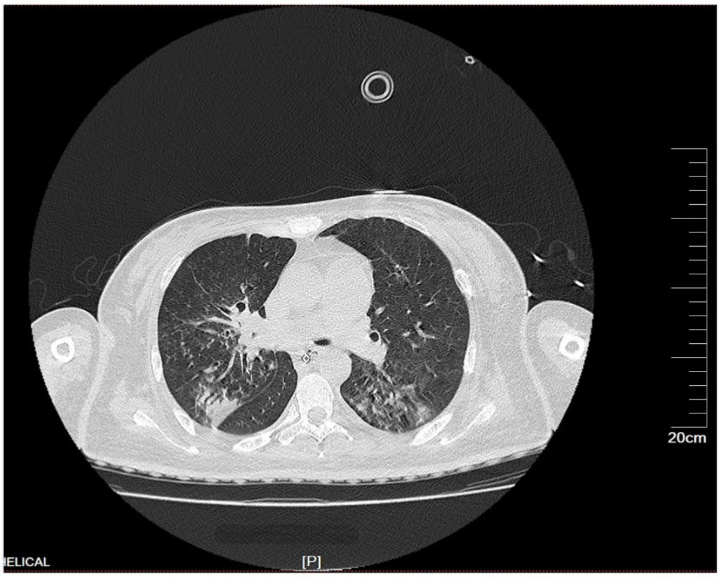

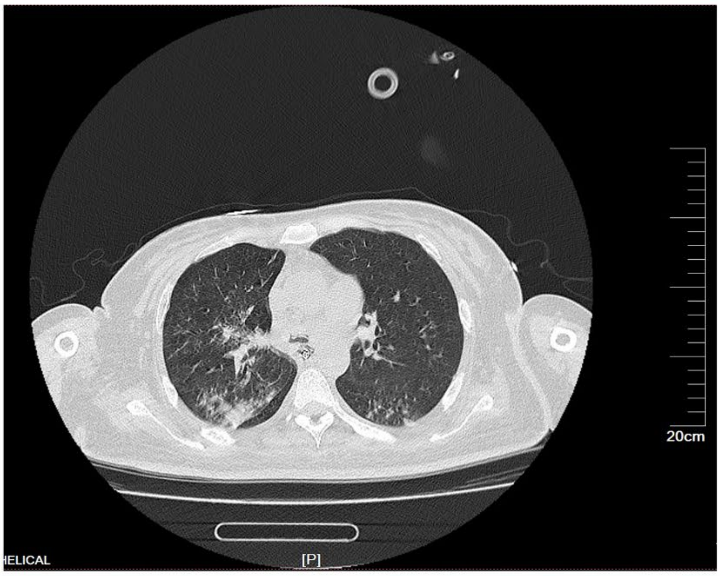

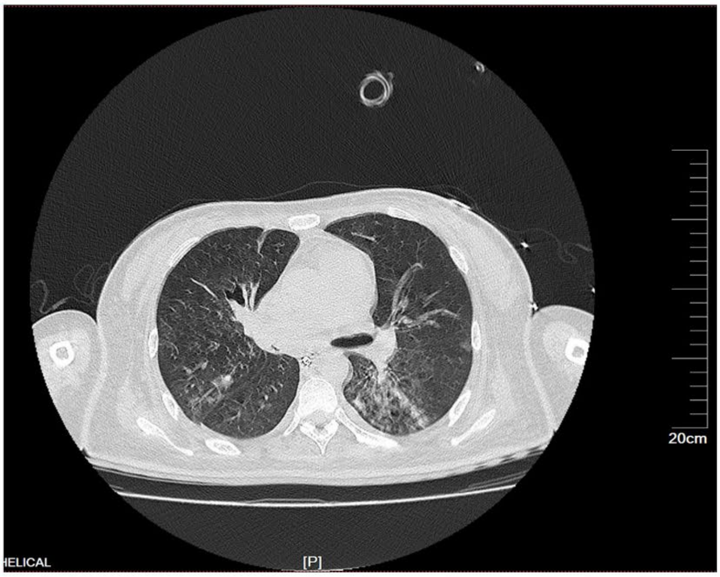

Hospital Day29

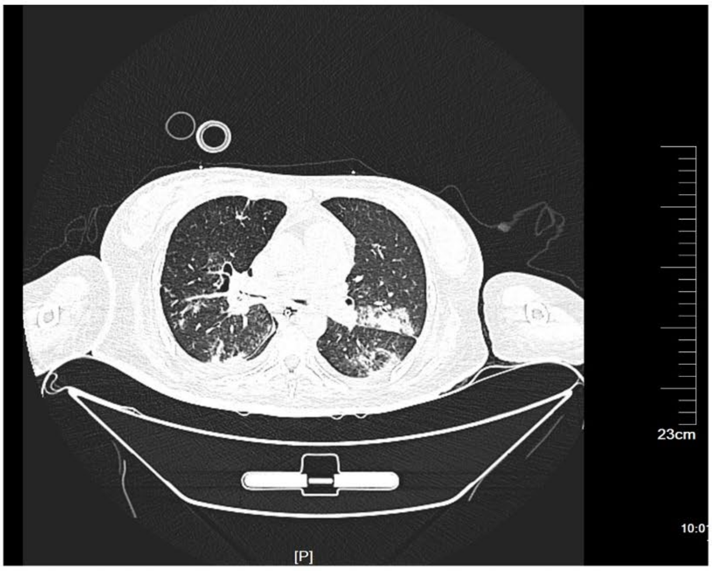

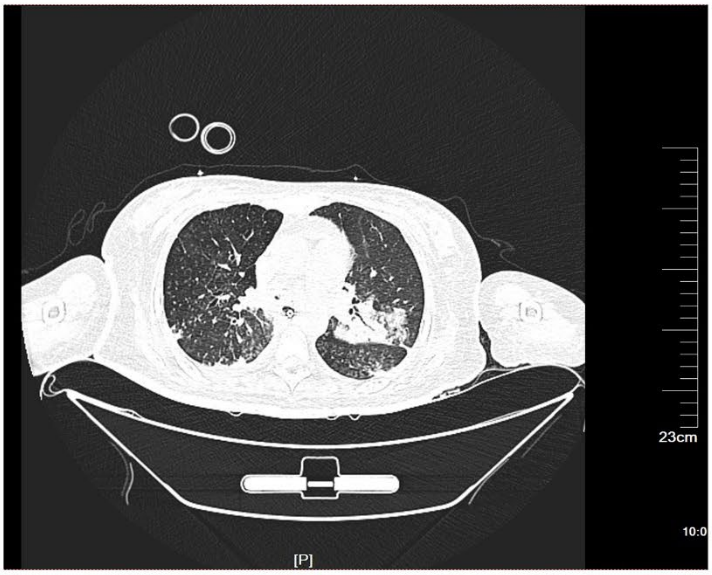

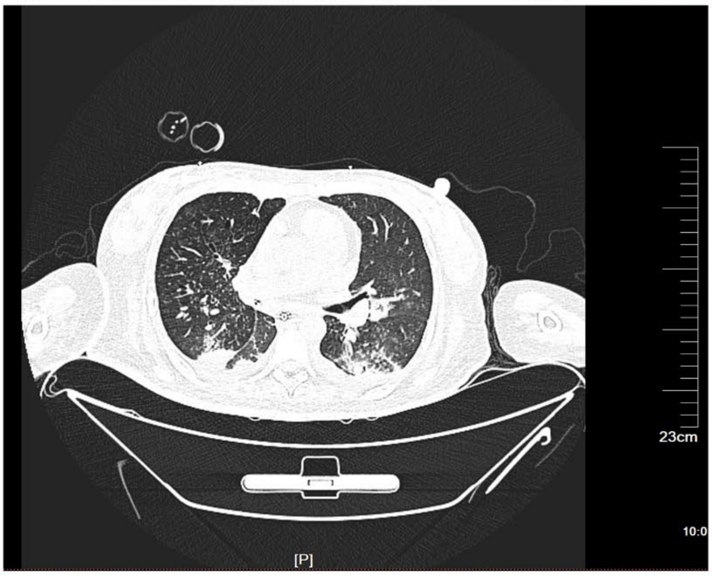

Hospital Day78

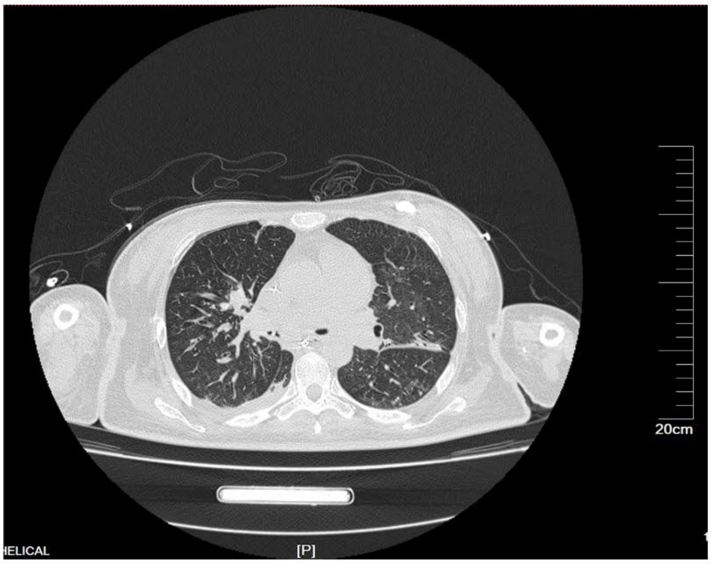

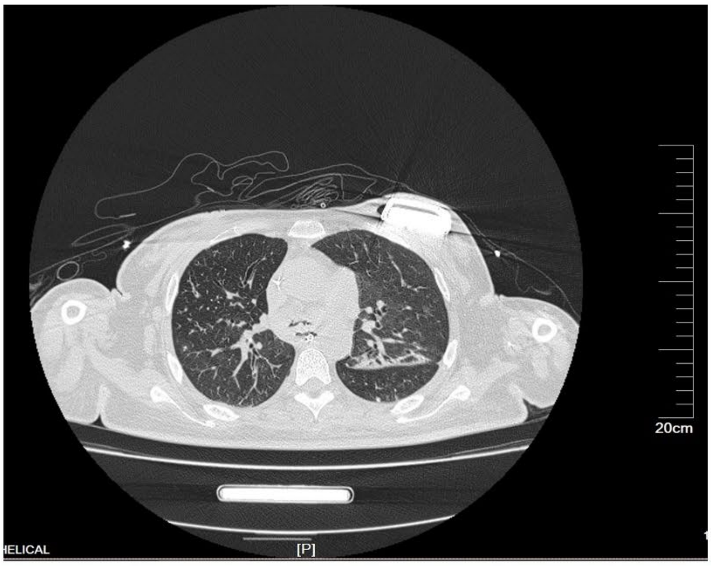

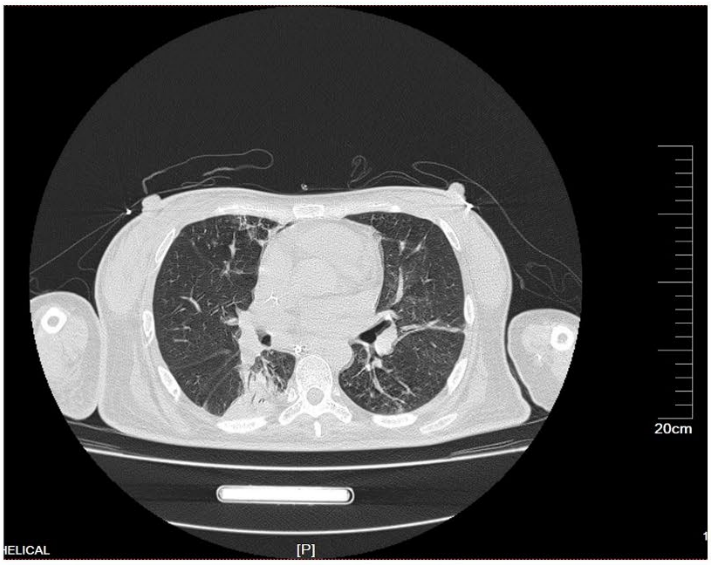

Bronchoscopy

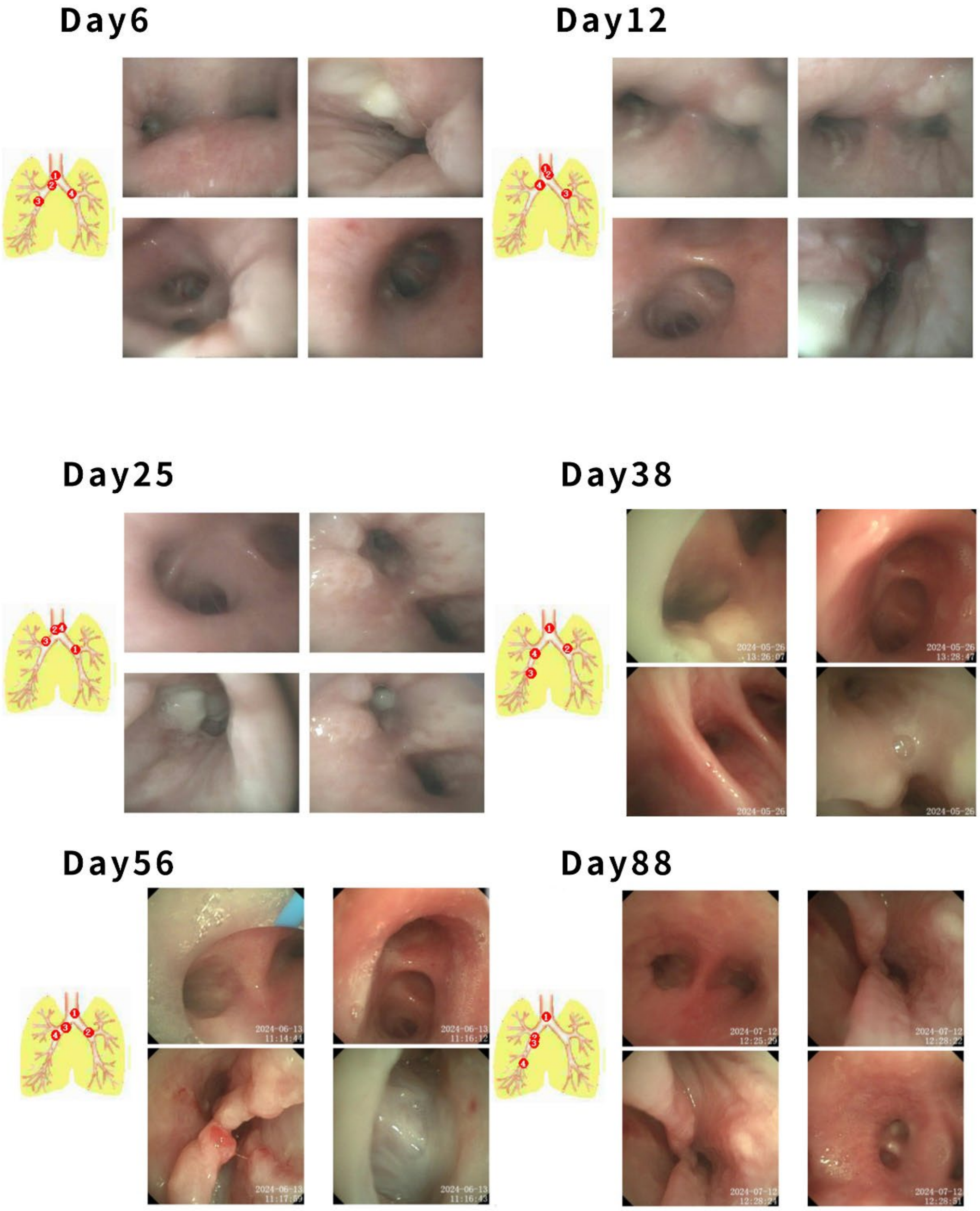

Expanded image of skin pallor

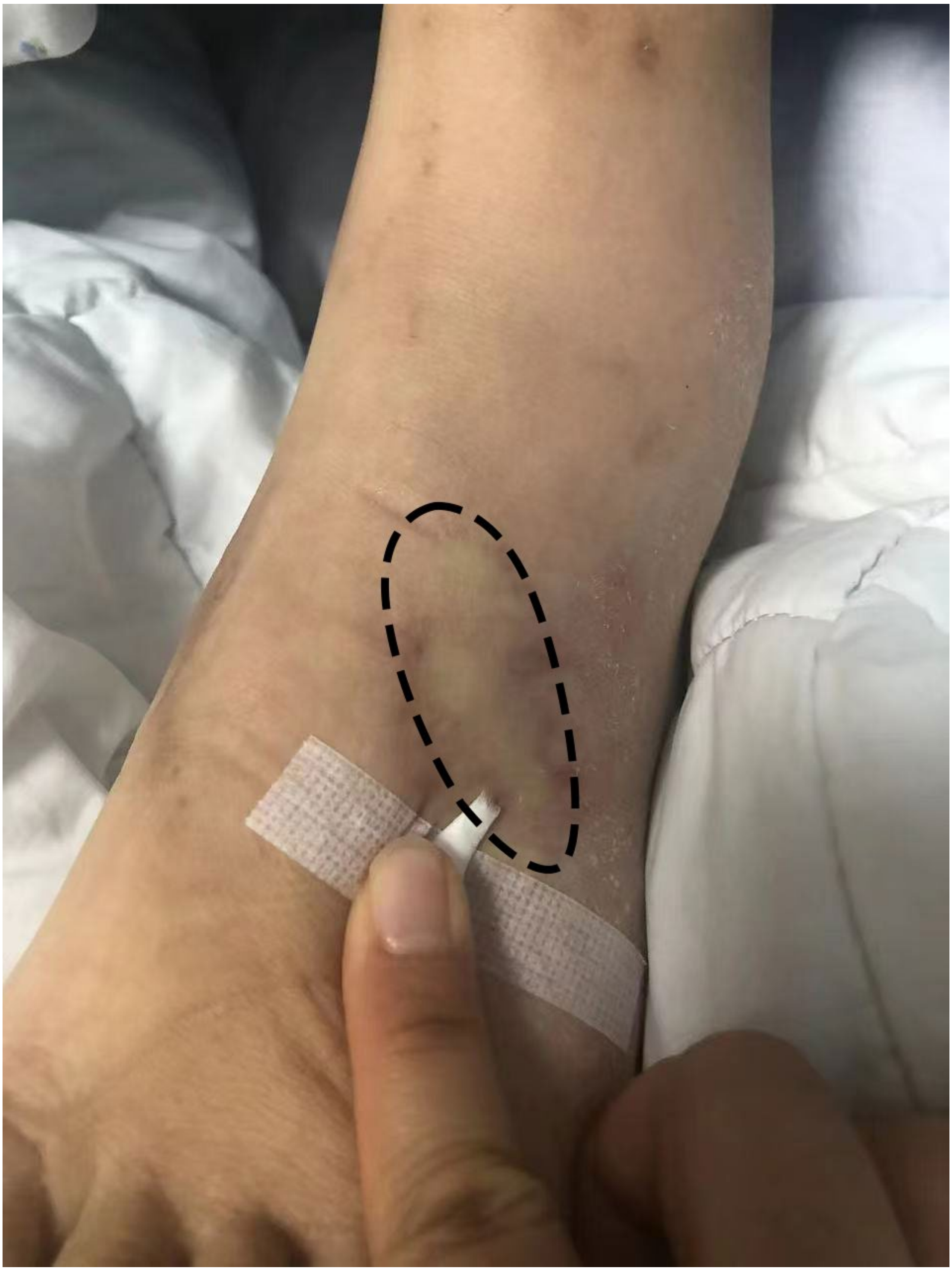

Supplement: Supplementary file 1 [file Data_Sheet_1.pdf]
